# Growth hormone-releasing hormone (GHRH) polymorphisms associated with carcass traits of meat in Korean cattle

**DOI:** 10.1186/1471-2156-7-35

**Published:** 2006-06-03

**Authors:** Hyun Sub Cheong, Du-Hak Yoon, Lyoung Hyo Kim, Byung Lae Park, Yoo Hyun Choi, Eui Ryong Chung, Yong Min Cho, Eng Woo Park, Il-Cheong Cheong, Sung-Jong Oh, Sung-Gon Yi, Taesung Park, Hyoung Doo Shin

**Affiliations:** 1 Department of Genetic Epidemiology, SNP Genetics, Inc., Seoul, 153-803, Korea; 2 National Livestock Research Institute, RDA, 441-706, Korea; 3 Department of Biotechnology, Sangi University, Wonju, Kangwon Do, 220-702, Korea; 4 Department of Statistics, Seoul National University, Seoul, 151-747, Korea

## Abstract

**Background:**

Cold carcass weight (CW) and longissimus muscle area (EMA) are the major quantitative traits in beef cattle. In this study, we found several polymorphisms of growth hormone-releasing hormone (GHRH) gene and examined the association of polymorphisms with carcass traits (CW and EMA) in Korean native cattle (Hanwoo).

**Results:**

By direct DNA sequencing in 24 unrelated Korean cattle, we identified 12 single nucleotide polymorphisms within the 9 kb full gene region, including the 1.5 kb promoter region. Among them, six polymorphic sites were selected for genotyping in our beef cattle (*n *= 428) and five marker haplotypes (frequency > 0.1) were identified. Statistical analysis revealed that -*4241A>T *showed significant associations with CW and EMA.

**Conclusion:**

Our findings suggest that polymorphisms in *GHRH *might be one of the important genetic factors that influence carcass yield in beef cattle. Sequence variation/haplotype information identified in this study would provide valuable information for the production of a commercial line of beef cattle.

## Background

The successful application of marker-assisted selection in the commercial animal population will depend on the identification of genes, including identification of genes underlying quantitative traits, exploration of genetic polymorphisms that are involved in different phenotypes of quantitative traits, and understanding how these genes/polymorphisms interact with the environment or with other genes affecting economic traits.

The growth hormone (GH) is essential for post-natal growth and general metabolism, and also plays an important role in lactation. Current knowledge indicates that GH exerts a key influence in nutrient use [1], mammary development [2], and growth [3]. There have been several reports of association between quantitative traits in cattle, such as growth performance and carcass merit, and polymorphisms in the GH gene [4-6].

The regulation of GH synthesis and secretion is multifactorial, but the predominant regulators of GH are the hypothalamic hormones, GH-releasing hormone (GHRH), GH secretagogue (GHS), and somatostatin (SS) [7]. In spite of the functional importance of GHRH in the regulation of GH, only one PCR-restriction fragment-length polymorphism (RFLP) [8] has been reported in cattle.

In this study, we examined *GHRH *as one of candidate genes in meat production. We performed extensive screening of *GHRH *by direct sequencing to detect polymorphisms and examined genetic association with the carcass traits. Here, we present 12 polymorphisms identified in *GHRH *and the results of an association study with meat quantity in Korean native cattle (Hanwoo).

## Results and discussion

**Figure 1:**
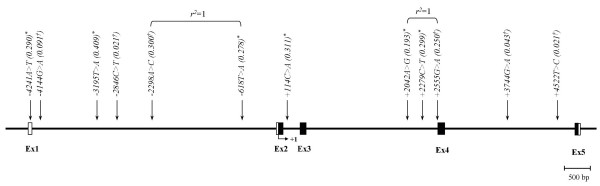
Map of SNPs in *GHRH *on chromosome 13. The exons are marked by black block, and 5' and 3' UTRs indicated by open blocks. First base of translational site is denoted as nucleotide +1. Asterisks (*) indicate polymorphisms genotyped in a larger Korean cattle (n = 428). ^†^The minor allele frequency based on 24 sequencing samples only, which is different with minor allele frequency of absolutely linked SNP genotyped in larger population.

By direct DNA sequencing, 12 polymorphisms were identified in *GHRH*: one in 5'UTR and 11 in introns. The locations and allele frequencies of polymorphism are shown in Table 1 and Figure 1. By pair-wise linkage analysis with DNA from the 24 unrelated Korean cattle, which were used for direct sequencing, we have found that two sets of polymorphisms were in absolute LDs (|D'| = 1 and *r*^2 ^= 1). Several sets of polymorphisms in complete LDs (|D'| = 1 and *r*^2 ^≠ 1) were also identified (Figure 1 and Table 2).

**Table 1:** Genotype and allele frequencies of 12 polymorphisms detected in *GHRH*

Name	Region	Genotype				Minor allele frequency	Heterozygosity	HWE*
-*4241A>T*	5'UTR	AA	AT	TT	N	0.290	0.412	0.903
					
		214^$^	180	34	428			
-*4144G>A*	Intron1	GG	AG	AA	N	0.091	0.165	0.896
					
		18	4	0	22			
-*3195T>A*	Intron1	TT	AT	AA	N	0.409	0.484	0.255
					
		140	222	63	425			
-*2846C>T*	Intron1	CC	CT	TT	N	0.021	0.041	0.995
					
		23	1	0	24			
-*2298A>C*	Intron1	AA	AC	CC	N	0.300	0.420	0.159
					
		8	12	0	20			
-*618T>A*	Intron1	TT	AT	AA	N	0.278	0.401	0.365
					
		217	183	27	427			
+*114C>A*	Intron2	CC	AC	AA	N	0.311	0.429	0.894
					
		199	186	39	424			
+*2042A>G*	Intron3	AA	AG	GG	N	0.193	0.311	0.968
					
		276	134	15	425			
+*2279C>T*	Intron3	CC	CT	TT	N	0.299	0.419	0.792
					
		206	184	35	425			
+*2555G>A*	Intron3	GG	AG	AA	N	0.250	0.375	0.777
					
		13	7	2	22			
+*3744G>A*	Intron4	GG	AG	AA	N	0.043	0.083	0.977
					
		21	2	0	23			
+*4522T>C*	Intron4	TT	CT	CC	N	0.021	0.041	0.995
					
		23	1	0	24			

* *P *value for deviation of genotype distribution from Hardy-Weinberg equilibrium (HWE)

^$^number of animals with that particular genotype

**Table 2:** Linkage disequilibrium coefficient (|*D*'| and *r*^2^) among *GHRH *SNPs

	*| D'|*						
	
	SNPs	-*4241A>T*	-*3195T>A*	-*618T>A*	+*114C>A*	+*2042A>G*	+*2279C>T*
*r*^2^	-*4241A>T*	-	0.550	0.121	0.935	0.835	0.129
	-*3195T>A*	0.175	-	0.928	1.000	0.748	0.545
	-*618T>A*	0.002	0.232	-	1.000	0.533	0.181
	+*114C>A*	0.160	0.317	0.174	-	0.941	0.126
	+*2042A>G*	0.404	0.187	0.026	0.094	-	1.000
	+*2279C>T*	0.016	0.089	0.029	0.015	0.100	-

Among identified polymorphisms, six SNPs (-*4241A>T, -3195T>A, -618T>A, +114C>A, +2042A>G *and +*2279C>T*) were selected for larger-scale genotyping based on LDs (only one polymorphism if there are absolute LDs (*r*^2 ^= 1)) and frequencies (> 0.1). Five major haplotypes (freq. > 0.1) were constructed (Table 3). Minor allele frequencies of SNP are shown in Table 1.

**Table 3:** Haplotypes and frequencies of *GHRH *among Korean native cattle.

Haplotype	-*4241A>T*	-*3195T>A*	-*618T>A*	+*114C>A*	+*2042A>G*	+*2279C>T*	Frequency
*ht1*	A	T	T	A	A	C	0.189
*ht2*	A	A	T	C	A	C	0.183
*ht3*	T	A	T	C	G	C	0.162
*ht4*	A	T	A	C	A	C	0.122
*ht5*	A	T	T	A	A	T	0.114
*ht6*	T	T	A	C	A	T	0.060
*ht7*	A	T	A	C	A	T	0.059
*ht8*	T	A	T	C	A	T	0.047
*ht9*	A	T	A	C	G	C	0.019
Others*	.	.	.	.	.	.	0.048

*Others contain rare haplotypes: AATCAT, AAACAC, TTTAAT, TTACGC, ATTCAT and TTACAC.

Associations of *GHRH *polymorphisms with carcass traits were analyzed using the mixed effect model with sire and age as covariates. Sire was treated as a random effect and age a fixed effect. The obtained *P *values were corrected for multiple testing by the effective number of independent marker loci (5.35) in *GHRH*. In addition, the permutation tests were performed for adjusting P values and for controlling the false discovery rate (FDR). Among five common haplotypes (freq. > 0.1) identified, *ht3 *was not used for further analysis because it was almost completely (> 93%) tagged by a single SNP, +*2042A>G *(Table 3). Haplotypes (*ht6 – ht9*) with frequencies less than 0.1 were not analyzed either.

By statistical analyses,-*4241A>T *showed significant associations with cold carcass weight (CW) and longissimus muscle area (EMA) and the genetic effects of -*4241A>T *were in gene dose dependent manner. CW and EMA were highest in "T" allele homozygotes (CW = 321.7 ± 36.4 and EMA = 78.2 ± 9.3), intermediate in "A/T" heterozygotes (CW = 315.3 ± 33.1 and EMA = 75.7 ± 8.7), and lowest in "A" allele homozygotes (CW = 306.2 ± 32.5 and EMA = 74.1 ± 8.2) (*P*^*COR *^= 0.025 and 0.046 for CW and EMA, respectively). The simple corrected *P*^*COR *^and the permutation based *P*^*WY *^provided consistent results, though there were some slight differences. The *Q*^*SAM *^represents the FDR value showing the consistent results. Similar mixed model was fit for the haplotype association analysis. However, none of the haplotypes were found to be significantly associated with CW and EMA (Table 4).

**Table 4:** Association analyses of the *GHRH *polymorphisms with carcass traits (CW and EMA) among Korean native cattle

Trait	Loci	Location	Genotype			*P*	*P*^*COR*^	*P*^*WY*^	*Q*^*SAM*^
							
			C/C*	C/R*	R/R*				
CW	-*4241A>T*	5'UTR	214(306.2 ± 32.5)	180(315.3 ± 33.1)	34(321.7 ± 36.4)	**0.005**	**0.025**	**0.025**	**0.025**
	-*3195T>A*	Intron1	140(306.6 ± 35.1)	222(312.6 ± 32.5)	63(314.6 ± 30.8)	0.449	1	0.952	1
	-*618T>A*	Intron1	217(311.9 ± 30.9)	183(310.6 ± 36.3)	27(309.9 ± 34.7)	0.859	1	0.995	0.859
	+*114C>A*	Intron2	199(314.2 ± 34.5)	186(308.8 ± 31.9)	39(309.5 ± 35.7)	0.496	1	0.952	1
	+*2042A>G*	Intron3	276(310.0 ± 35.2)	134(313.6 ± 29.6)	15(317.8 ± 29.1)	0.835	1	0.995	1
	+*2279C>T*	Intron3	206(308.7 ± 32.4)	184(313.3 ± 32.8)	35(316.9 ± 40.9)	0.815	1	0.995	1
	*ht1*	.	282(313.5 ± 33.1)	129(306.6 ± 34.0)	16(308.9 ± 34.2)	0.219	1	0.474	0.377
	*ht2*	.	285(311.8 ± 33.9)	128(309.3 ± 32.1)	14(316.3 ± 36.9)	0.594	1	0.509	0.554
	*ht4*	.	330(312.5 ± 32.5)	90(307.3 ± 37.3)	7(302.4 ± 20.1)	0.174	0.931	0.474	0.59
	*ht5*	.	332(311.2 ± 34.0)	93(311.6 ± 32.0)	2(299.5 ± 29.0)	0.340	1	0.474	0.401

EMA	-*4241A>T*	5'UTR	214(74.1 ± 8.2)	180(75.7 ± 8.7)	34(78.2 ± 9.3)	**0.009**	**0.046**	0.064	0.066
	-*3195T>A*	Intron1	140(75.2 ± 7.6)	222(75.0 ± 8.9)	63(75.6 ± 9.6)	0.583	1	0.991	1
	-*618T>A*	Intron1	217(75.2 ± 8.2)	183(75.2 ± 9.1)	27(74.1 ± 7.7)	0.742	1	0.998	1
	+*114C>A*	Intron2	199(75.1 ± 9.5)	186(75.0 ± 7.8)	39(75.3 ± 6.4)	0.945	1	0.998	0.95
	+*2042A>G*	Intron3	276(75.1 ± 8.5)	134(75.1 ± 8.9)	15(76.8 ± 9.0)	0.799	1	0.998	1
	+*2279C>T*	Intron3	206(74.3 ± 9.3)	184(75.6 ± 7.6)	35(77.5 ± 8.6)	0.920	1	0.998	1
	*ht1*	.	282(75.2 ± 8.8)	129(75.0 ± 8.3)	16(76.1 ± 5.9)	0.716	1	0.713	0.671
	*ht2*	.	285(75.6 ± 8.2)	128(74.3 ± 9.1)	14(74.3 ± 9.9)	0.402	1	0.574	0.486
	*ht4*	.	330(75.7 ± 8.3)	90(73.7 ± 9.3)	7(68.3 ± 6.0)	0.022	0.12	0.088	0.089
	*ht5*	.	332(75.2 ± 8.9)	93(74.9 ± 7.1)	2(76.0 ± 2.8)	0.316	1	0.528	0.572

Genotype and haplotype distributions, means, standard deviations (SD), *P *values controlling for sire and age at slaughter as covariates was shown. *C/C, C/R, and R/R represent the common allele, heterozygotes and homozygotes for the rare allele, respectively. To achieve a simple correction for multiple testing of single-nucleotide polymorphisms (SNPs) in linkage disequilibrium (LD) with each other, the effective number of independent marker loci (5.35) in *GHRH *was calculated using the software SNPSpD [23], on the basis of the spectral decomposition (SpD) of matrices of pair-wise LD between SNPs [24]. *P*^*COR *^represents the simple corrected *P *value. The permutation based *P *values, *P*^*WY *^were obtained by the Westfall and Young's method [25]. The FDR values *Q*^*SAM *^were estimated using the permutation test [26].

Linear growth in vertebrate organisms is highly dependent on the GH [9]. GH is a lipolytic hormone, activating lipase and thereby mobilizing fat from adipose tissue [10]. While the primary action of GH is to stimulate skeletal and visceral growth, it has important metabolic actions as well. As a consequence of these actions, deficiency of GH can result in lowered growth rate [11], delayed bone maturation [12], decreased body mass [13], and hypoglycemia [14].

In a recent study, a *GHRH *polymorphism (*Alu*I) was associated with yield traits (the average daily gain and expected progeny difference for fat thickness) in landrace pigs [15]. In this study, we also found that polymorphisms in *GHRH*, one of the predominant regulators of GH releasing [16], were associated with carcass yield traits (CW and EMA) in Korean native cattle.

Although the mechanisms involved in the association of alternative genotypes in the UTR and intronic SNPs with CW and EMA are not currently understood, the crucial role of the non-coding portion of genomes is now widely acknowledged. Polymorphisms within introns can affect gene function by affecting both the splice donor or acceptor site or regions nearby and regulatory motifs within introns. And UTRs are involved in many post-transciptional regulatory pathways that control mRNA localisation, stability, translation efficiency and initiation of protein synthesis. The post-transcriptional events play an important, yet incompletely understood, role in regulatory gene expression and cellular behaviour; many of the identified *cis*-acting elements for translational regulation occur within the UTR [17].

## Conclusion

We have identified 12 polymorphisms in *GHRH*, and six polymorphic sites were selected for genotyping in Korean native cattle. Statistical analysis revealed that *GHRH -4241A>T *showed significant association with carcass traits. Replication of our finding in an independent dataset and/or functional validation of polymorphisms should be performed in the future.

## Methods

### Animals and phenotypic data

The Korean native cattle genomic DNA samples were obtained from 428 steers produced from 76 sires used in progeny testing program of National Livestock Research Institute (NLRI) of Korea. The dams were inseminated randomly with young sires. All steers were fed for 731.39 ± 16.53 days period under tightly controlled feeding program in Daekwanryeong and Namwon branch of NLRI. Live weights were determined before slaughter. Mean of live weights was 539.93 ± 51.96 kg. Yield grades for carcasses were determined by CW and EMA. After a 24-h chill, CW was measured, and then the left side of each carcass was cut between the last rib and the first lumbar vertebrae to determine EMA. EMA was determined at the surface using a grid [18]. Means of carcass traits were 311.47 ± 33.39 kg (CW) and 75.16 ± 8.62 cm^2 ^(EMA).

### Sequencing analysis of GHRH

We have sequenced the 9 kb full genome, including the promoter region (1.5 kb), to discover variants in 24 unrelated Korean native cattle using the ABI PRISM 3730 DNA analyzer (Applied Biosystems, Foster City, CA). Nineteen primer sets for the amplification and sequencing analysis were designed based on GenBank sequences (Ref. Genome seq.; AF242855 released on July 30, 2000). Primer information is available on website [19]. Sequence variants were verified by chromatograms.

### Genotyping by single-base extension (SBE) and electrophoresis

For genotyping of polymorphic sites, amplifying and extension primers were designed for single-base extension (SBE) [20]. Primer extension reactions were performed with the SNaPshot ddNTP Primer Extension Kit (Applied Biosystems). To clean up the primer extension reaction, one unit of SAP (shrimp alkaline phosphatase) was added to the reaction mixture, and the mixture was incubated at 37°C for 1 hour, followed by 15 min at 72°C for enzyme inactivation. The DNA samples, containing extension products, and Genescan 120 Liz size standard solution was added to Hi-Di formamide (Applied Biosystems) according to the recommendations of the manufacturer. The mixture was incubated at 95°C for 5 min, followed by 5 min on ice, and then electrophoresis was performed using the ABI Prism 3100 Genetic Analyzer. The results were analyzed using the program of ABI Prism GeneScan and Genotyper (Applied Biosystems). Probe information is available on website [19].

### Statistics

The X^2 ^tests were used to determine whether individual variants were in equilibrium at each locus in the population (Hardy-Weinberg equilibrium). We examined a widely used measure of linkage disequilibrium between all pairs of biallelic loci, Lewontin's D' (|D'|) [21], and *r*^2^. Haplotypes and their frequencies were inferred using the algorithm developed by Stephens et al [22]. Phase probabilities of each site were calculated for each individual using this software. Association analyses with carcass traits (CW and EMA) were performed using a mixed effect model treating "sire" as a random effect. Age at slaughter was also included in the model. Other covariates were not available for this analysis. We fit a full model that includes all six SNPs in the model. We think the full model is more appropriate for controlling the closely linked SNPs more effectively. The effective number of independent marker loci in *GHRH *was calculated to correct for multiple testing. The effective number in *GHRH *was calculated using the software SNPSpD [23], which is based on the spectral decomposition (SpD) of matrices of pair-wise LD between SNPs. The resulting number of independent marker loci was applied to correct for multiple testing [24]. In addition, we performed the permutation test by controlling the P values by the Westfall and Young's method [25] and by controlling the FDR [26]. For the haplotype analyses, we fit the model including four haplotypes with the same covariates and performed the permutation test in a similar manner.

## Authors' contributions

HS Cheong and HD Shin carried out the statistical analyses and drafted the manuscript. LY Kim, BL Park and YH Choi carried out the sequencing, SNP discovery and genotyping. DH Yoon, ER Chung and EW Park participated in the animal and phenotypic data collection. YM Cho participated in the design of the study. IC Cheong and SJ Oh participated in its design and coordination and helped to draft the manuscript. SG Yi and T Park carried out further statistical analyses. All authors read and approved the final manuscript.
